# Correction: Assessing the emergence time of SARS-CoV-2 zoonotic spillover

**DOI:** 10.1371/journal.pone.0338468

**Published:** 2025-12-09

**Authors:** Stéphane Samson, Étienne Lord, Vladimir Makarenkov

After publication of this article [1], the following concerns were raised:

1. Figs 3A, 4A, and 5A show the virus RaTG13 sharing a common ancestor with other viruses sometime between 2015 and 2019 (tMRCA) but RaTG13 was sampled in 2013.
2. Fig 5A shows that MP789 appears to be as closely related to SARS-CoV-2 as BANAL-20–52, a statement that only makes sense if an amino acid alignment was used for the receptor binding domain analysis. This is not explained in the article.

After correspondence with the authors and assessment by a member of the *PLOS One* Editorial Board, the article’s overall results and conclusions are upheld, and the authors provide the following clarifications:

1. Due to visualization constraints, the figures themselves only show the median posterior estimates for all nodes, rather than the complete 95% highest posterior density (HDP) intervals. For RaTG13, those HPD 95% intervals include, as expected, dates prior to its 2013 sampling. The tMRCA is not the exact date of the spillover event. The August–October 2019 date range refers to the divergence time of the SARS‑CoV‑2 lineage from its closest sampled relatives contained in the two datasets. This divergence time is used as a proxy for the probable zoonotic spillover period since the datasets include both the closest known animal coronaviruses and post‑spillover variants.
2. The RBD dataset is an amino acid alignment, which explains certain topological patterns in Fig 5. While this methodological choice is visible in S5 Table and in the publicly available dataset files, it is not explicitly stated in the main methodology section.
